# Supplementary material for: Phenotypic and Genomic Characterization of ESBL- and AmpC-β-Lactamase-Producing Enterobacterales Isolates from Imported Healthy Reptiles
Source: Antibiotics (Basel). 2024 Dec 20;13(12):1230. doi: 10.3390/antibiotics13121230 (PMC11726957; doi:10.3390/antibiotics13121230)
Supplement: Supplementary file 1 [file antibiotics-13-01230-s001.zip › Table S1_MIC data.pdf]

Table S1. MIC values for 79 *Enterobacterales* isolates with interpretation by ECOFF according to EUCAST to differentiate between wild type and non-wild type

		MIC values in mg/L											WT	nWT	n total
**Antimicrobial**	**Species**	**0.03125**	**0.0625**	**0.125**	**0.25**	**0.5**	**1**	**2**	**4**	**8**	**16**	**32**			
Amoxi/Clav	*E. coli*							2	7	10	‖7	16	19	23	42
	*Klebsiella**								1	3	‖3	4	4	7	11
	Citrobacter+										1	11			12
	Enterobacter#										‖	1		1	1
Ampicillin	*E. coli*										‖	42	0	42	42
	Klebsiella											13		IR	13
	Citrobacter										1	11		IR	12
	Enterobacter#										‖	1		1	1
Ceftiofur	*E. coli*						1	‖	3	38			1	41	42
	Klebsiella					1	1	‖	1	10			2	11	13
	Citrobacter						1	‖2	1	8			1	11	12
	Enterobacter						3	‖2	4	3			3	9	12
Colistin	*E. coli*					25	14	1	‖2				40	2	42
	Klebsiella					9	4		‖				13		13
	Citrobacter					5	5	2	‖				12		12
	Enterobacter					4	1	2	‖5				7	5	12
Enrofloxacin	*E. coli*	1	8		‖3	6	8	16					9	33	42
	Klebsiella	2	3		‖	2	2	4					8	5	13
	Citrobacter	2	1		‖4	1		4					3	9	12
	Enterobacter	4	4	1	‖	2		1					9	3	12
Florfenicol[1]	*E. coli*								14	17	10	‖			42
	Klebsiella							1	9	1	‖2		11	2	13
	Citrobacter								1	6	5	‖			12
	Enterobacter							2	3	6	2	‖			12
Gentamicin	*E. coli*						2	32	‖		8		34	8	42
	Klebsiella						4	6	‖	3			10	3	13
	Citrobacter						5	5	‖		2		10	2	12
	Enterobacter						6	5	‖		1		11	1	12
Tetracycline	*E. coli*							7			‖35		7	35	42
	Klebsiella						3	3			‖7		6	7	13

Antimicrobial	Species	MIC values in mg/L 0.03125	0.0625	0.125	0.25	0.5	1	2	4	8	16	32	WT	nWT	n total
	Citrobacter						2	5			‖5		7	5	12
	Enterobacter							2	6	1	‖3		9	3	12
Trim/Sulf	*E. coli*				15	3	‖1	1	22				18	24	42
	Klebsiella			1	4		‖		8				5	8	13
	Citrobacter			5	2		‖		5				7	5	12
	Enterobacter			4	5		‖		3				9	3	12

‖ marks the distinction from wild type to non-wild type according to ECOFFs by EUCAST (https://mic.eucast.org/). When values for certain antimicrobials were not available for a species, the values for *E. coli* were used, taking into account known intrinsic resistance as recommended in CLSI [29]. WT: wild type; nWT: non wild type; IR: intrinsically resistant; Amoxi/Clav: amoxicillin/clavulanic acid (AMC; 2:1); Trim/Sulf: trimethoprim/sulfamethoxazole (1:19). Concentrations that have not been tested in the layout are highlighted in grey. The white fields indicate the concentrations tested, and if the value is outside the white fields, the value obtained was above or below the highest or lowest concentration tested. Due to interim changes to the concentration levels in the Micronaut layout, high concentration levels were omitted and low concentration levels were added. The points of concentration, which were the same in all layouts, are shown.

* Since *K. aerogenes* is intrinsically resistant to AMC, only *K. pneumoniae* is shown here.

+ Intrinsic resistance to AMC is different for several *Citrobacter* species. Since the *Citrobacter* spp. isolates shown in this study often could not be reliably differentiated to species level, no interpretation as wild type or non-wild type is given for AMC.

# Since *Enterobacter cloacae* complex is intrinsically resistant to AMP and AMC, only *E. soli* is shown in these columns. However, little is known about resistance in *E. soli*, so interpretation should be assessed with caution.

Due to intrinsic resistance, no results are shown for erythromycin, tilmicosin, penicillin and tiamulin (CLSI VET01S ED7:2024 Appendix B). MIC levels were high in all 79 isolates for these antimicrobials.

[1] Note: Florfenicol: highest concentration in the Micronaut layout is 8 mg/L, ECOFF is 16, therefore no classification into WT and non-WT is possible, except for *Klebsiella* spp.
