# Supplementary material for: Phenotypic and Genomic Characterization of ESBL- and AmpC-β-Lactamase-Producing Enterobacterales Isolates from Imported Healthy Reptiles
Source: Antibiotics (Basel). 2024 Dec 20;13(12):1230. doi: 10.3390/antibiotics13121230 (PMC11726957; doi:10.3390/antibiotics13121230)
Supplement: Supplementary file 1 [file antibiotics-13-01230-s001.zip › Table S3_Primer.pdf]

Table S3: Oligonucleotide primers used to generate DIG-Oxigenin-labeled probes in order to determine the location of ESBL and AmpC-β-lactamase genes on plasmid or chromosome

Primer name	Target genes	5´- 3´sequence	Amplicon length	Reference
*bla*SHV fw	*bla*$_{SHV}$-like	GGGTTATTCTTATTTGTCGC	928 bp	[88]
*bla*SHV rev		TTAGCGTTGCCAGTGCTC		
DHAM_F	*bla*$_{DHA-1}$, *bla*$_{DHA-2}$	AACTTTCACAGGTGTGCTGGGT	405 bp	[89]
DHAM_R		CCGTACGCATACTGGCTTTGC		
CITM_F	*bla*$_{CMY-2}$ to *bla*$_{CMY-7}$; *bla*$_{LAT-1}$ to *bla*$_{LAT-4}$	TGGCCAGAACTGACAGGCAAA	462 bp	[89]
CITM_R		TTTCTCCTGAACGTGGCTGGC		
M13U CTX-M 1F	*bla*$_{CTX-M}$ group 1	GGTTAAAAAATCACTGCG	977 bp	This study
CTX-M R		GAGCTTATGGCCTGGTAT		
CTX-M group 2 fw	*bla*$_{CTX-M}$ group 2	ATGATGACTCAGAGCATTCGC	876 bp	This study
CTX-M group 2 rv		TCAGAAACCGTGGGTTACGAT		
CTX-M-9 fw	*bla*$_{CTX-M}$ group 9	GTGACAAAGAGAGTGCAACGG	900 bp	[90]
CTX-M-9 rev		ATGATTCTCGCCGCTGAAGCC		

References

88. Rasheed, J.K.; Jay, C.; Metchock, B.; Berkowitz. F.; Weigel, L.; Crellin, J.; Steward, C.; Hill, B.; Medeiros, A.A.; Tenover, F.C. Evolution of extended-spectrum beta-lactam resistance (SHV-8) in a strain of *Escherichia coli* during multiple episodes of bacteremia. *Antimicrob Agents Chemother* 1997, *41*, 647-653. doi: 10.1128/AAC.41.3.647.

89. Pérez-Pérez, F.J.; Hanson, N.D. Detection of plasmid-mediated AmpC beta-lactamase genes in clinical isolates by using multiplex PCR. *J Clin Microbiol* 2002, *40*, 2153-62. doi: 10.1128/JCM.40.6.2153-2162.2002.

90. Cantón, R.; Oliver, A.; Coque, T.M.; del Carmen Varela, M.; Pérez-Diaz, J.C; Baquero, F. Epidemiology of Extended-Spectrum β-Lactamase-Producing *Enterobacter* Isolates in a Spanish Hospital during a 12-Year Period. *J Clin Microbiol* **2002**, *40*, 1237-1243, doi: 10.1128/JCM.40.4.1237-1243.2002.
